# Influence of Sequential Liquid Ammonia and Caustic Mercerization Pre-Treatment on Dyeing Performance of Knit Cotton Fabric

**DOI:** 10.3390/ma15051758

**Published:** 2022-02-25

**Authors:** Lina Lin, Tiancheng Jiang, Yonghong Liang, Md. Nahid Pervez, Rahul Navik, Bo Gao, Yingjie Cai, Mohammad Mahbubul Hassan, Naveeta Kumari, Vincenzo Naddeo

**Affiliations:** 1 Hubei Provincial Engineering Laboratory for Clean Production and High-Value Utilization of Bio-Based Textile Materials, Colllege of Chemistry and Chemical Engineering, Wuhan Textile University, Wuhan 430200, China; linalin@wtu.edu.cn (L.L.); 2015383113@mail.wtu.edu.cn (T.J.); 1815073028@mail.wtu.edu.cn (Y.L.); mpervez@unisa.it (M.N.P.); rahul.navik2012@gmail.com (R.N.); 2 Engineering Research Centre for Clean Production of Textile Dyeing and Printing, Ministry of Education, College of Environmental Engineering, Wuhan Textile University, Wuhan 430200, China; 3 Sanitary Environmental Engineering Division (SEED), Department of Civil Engineering, University of Salerno, 84084 Fisciano, Italy; 4 College of Art and Design, Wuhan Textile University, Wuhan 430200, China; 5 Fashion, Textiles and Technology Institute, University of the Arts London, London W1G 0BJ, UK; mahbubul.hassan@arts.ac.uk; 6 School of Fashion and Textiles, Brunswick Campus, RMIT University, Melbourne, VIC 3001, Australia; naveeta.kumari@rmit.edu.au

**Keywords:** caustic mercerization, liquid ammonia, pre-treatment, soft handle, reactive dyeing

## Abstract

A two-stage sequential pretreatment including caustic mercerization (CM) and liquid ammonia (LA) treatment was applied to investigate the influence on dyeing performance and handle of knit cotton fabric, and the relationship between dye size and dyeing properties. Various techniques were applied to characterize all the treated fabrics. X-ray diffraction (XRD) and Fourier-transform infrared (FTIR) analyses of the treated fabrics confirmed that both sequential treatments decreased the crystallinity of cotton fabric more than only the CM or LA treatment. The pattern of cellulose I was transferred to a mixed configuration of cellulose II and cellulose III after the CM/LA or LA/CM treatment. Thermal performances measured by thermogravimetric analysis (TGA) and differential thermogravimetry (DTG) techniques showed that the thermal stability of the treated cotton only marginally decreased. The wicking height increased after the sequential CM/LA treatment, indicating that the hydrophilicity of the fabric increased. The dye absorption and color uniformity were better for the reactive dye with a smaller molecular weight (Reactive Red 2) compared with the one with a larger molecular weight (Reactive Red 195). The total dye fixation efficiency (T%) increased to 72.93% and 73.24% for Reactive Red 2 dyeings of CM/LA- and LA/CM-cotton fabric from 46.75% of the untreated fabric, respectively; the T% increased to 65.33% and 72.27% for Reactive Red 195 dyeings of CM/LA- and LA/CM-cotton fabric from 35.17% of the untreated fabric, respectively. The colorfastness and dye exhaustion and fixation percentages of the samples were enhanced after the treatments. Furthermore, compared to the single CM or LA treatment, the softness handle properties were further improved after the fabrics were sequentially treated by CM/LA. The developed pre-treatment of CM/LA can be used in the textile industry to promote the dyeability, handle, and mechanical properties of knit cotton fabrics.

## 1. Introduction

Mercerization pretreatment by caustic mercerization (CM) or liquid ammonia (LA) in the textile sector is often employed to improve several characteristics of cotton fabrics, including luster, tensile strength and dyeability [1,2]. In contrast with CM treatment, the advantage of LA treatment is cleaner because liquid ammonia is highly volatile and can be completely recycled after mercerization [3,4,5]. After CM treatment, the Na^+^ ions inside the cotton fiber are hard to wash off, and it critically influences the dyeing performance without the efficient removal of Na^+^ ions. It is therefore important to wash the CM-treated cotton fabric multiple times in both hot and cold water. The defect of LA treatment is its higher treating cost, compared with that of CM. Another distinction between CM and LA treatments is the contribution to fabric handle. Liquid ammonia treatment could have an improved handle and softness [6,7], while caustic mercerization gives the woven fabric a stiff handle [8,9]. 

It is known that mercerization by CM or LA decreases the crystallinity of cotton fiber, i.e., more amorphous regions in fiber are produced, but LA treatment is more effective than CM [10]. The crystallinity of untreated cotton fiber was 65.7%, and it decreased to 61.4% and 58.6% by CM and LA treatment, respectively [6]. Thus, the dyeability of mercerized cotton fiber should increase based on the dyeing principle. However, occasional reports from the dyeing plant claimed that the LA treatment did not enhance the dyeing performance of the cotton fabric, and even becomes poorer than the untreated one. The phenomena were confirmed by Wakida et al. research [6]. The woven cotton fabric treated by LA had a similar dye uptake of Direct Blue 1 dye with that of the untreated fabric. Therefore, there is a conflict between the crystallinity decrease and the poorer dyeability. This conflict is explained in that both treatments enlarged the cumulative accessible pore volume, which expressed an increase of the amorphous region. However, the CM treatment expands the fiber pores, while the LA treatment compacts the fiber pores. Thus, the LA-treated cotton fiber only exhibits a poorer dyeability in using big reactive dyes [11].

The influence of a two-step of CM/LA and LA/CM pre-treatment on dyeability and handle of woven cotton fabric was reported [6,7], which woven cotton fabric was dyed with direct dye (Direct Blue 1) and the handle was measured by shearing modulus and bending hysteresis that obtained with a KES instrument. Recently, LA pre-treatment of knit cotton fabric is a burgeoning market. Meanwhile, in exhaust dyeing of knit cotton fabric, bi-functional reactive dyes (almost all of the dyes are big molecules) are mainly applied. The dyeing mechanism of cotton fiber with reactive dye differs from the direct dye. The former is chemical absorption, while the latter is physical adsorption. Hence, without any dye fixation post-treatment, the reactive dyed cotton fiber shows a higher colorfastness to washing and rubbing, compared to direct dyed one. Therefore, it is significant to investigate the dyeing performance and handle of CM, LA, CM/LA, and LA/CM pre-treated knit cotton fabric with reactive dye.

In the present work, a treatment process was designed in which knit cotton fabrics were treated by CM, LA, CM/LA, and LA/CM processes. The fabrics were subsequently tested for their wicking, dyeing, and handle properties. Two types of dyes, including Reactive Red 2 (low molecular weight) and Reactive Red 195 (high molecular weight) were used to determine the relationship between dye size and dyeing properties, based on the exhaustion, fixation and total dye fixation of reactive dyes. Besides, fabric softness was directly obtained with a WOOL HandleMeter instrument.

## 2. Materials and Methods

### 2.1. Materials

**Figure 1:**
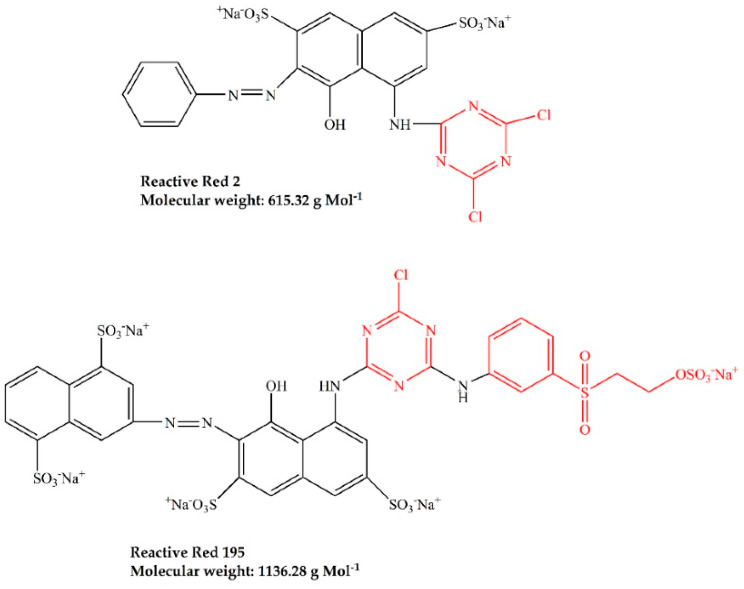
Chemical structures and molecular weights of Reactive Red 2 and Reactive Red 195.

Commercial unbleached 100% knit cotton fabric (160 g m^−2^) was obtained from TST Group Holding, Ltd. (Guangzhou, China) Both dyes of Reactive Red 195 (Red 195) and Reactive Red 2 (Red 2) were supplied by Shanghai Jiaying Chemical Company (Shanghai, China), characteristics of which are depicted in Figure 1. Anhydrous Liquid ammonia was supported by Wuhan Niuruide Gas Company (Wuhan, China). Commercial grade 500 Luton non-ionic detergent Luton 500 was purchased from Dalton UK Company (Shanghai, China).

### 2.2. Sequential Treatment

The knit cotton fabrics were treated with tension in all experiments. For the caustic mercerization (CM) treatment, a piece of knit cotton fabric sample was treated in 250 g L^−1^ NaOH solution at 23 °C for 3 min and then washed by warm water (about 60 °C) for 10 min, followed by cold water, then by HCL with a concentration of 2 g L^−1^ at 23 °C for 10 min, and finally through cold water again until neutral. 

The liquid ammonia (LA) treatment involved immersing knit cotton fabric samples in anhydrous liquid ammonia at −40 °C for 3 min. Subsequently, the sample was dried at 70 °C for 20 min. Finally, the residual ammonia was removed by washing with cold water, followed by 2 g L^−1^ HCl at 23 °C for 10 min, and then by cold water until neutral. 

The two-stage CM/LA treatment or the two-stage LA/CM was conducted following the procedure mentioned above.

### 2.3. Characterization

#### 2.3.1. XRD Analysis

Using an X-ray diffractometer (Rigaku Ultima III, Tokyo, Japan), the powder XRD patterns of the untreated and treated fabrics were determined. The crystal phase formation was scanned from 2θ = 5–60° having step size of 0.02° with CuKα radiation (λ = 1.54056 Å) [12] source. 

#### 2.3.2. FTIR Analysis

Fourier Transformation Infrared (FTIR) spectra of the untreated and treated samples were evaluated using a Bruker Optik EQUINOX 55 spectrophotometer (Ettlingen, Germany). The wavenumber range was 4000–400 cm^−1^. Samples were cut and mixed with potassium bromide, and then ground to prepare a pellet that was stored at room temperature (23 °C).

#### 2.3.3. Thermogravimetric Analysis

The thermal stabilities were carried out by a thermogravimetric analyzer (TGA/DSC1, Mettler-Toledo, LLC, Shanghai, China). The curves were observed under nitrogen atmosphere (flow rate of 50 mL min^−1^) by maintaining a heating rate of 10 °C min^−1^ in the range of 30–700 °C.

#### 2.3.4. Wicking Height

The knit cotton fabric was prepared to 25 cm (warp direction) × 3 cm (weft direction), and the liquid bath contained 5% (*w*/*w*) of potassium bichromate. Measurements were made as per the FZ/T 01071-2008 standard with a textile capillary effect tester (YG871, Ningbo Textile Instrument Factory, Ningbo, China). The liquid wicking height values were recorded at 1, 5, 10, 20, and 30 min. 

#### 2.3.5. Dyeing Process

**Figure 2:**
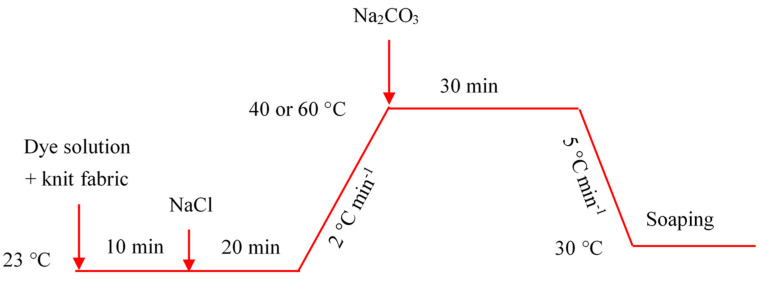
Dyeing methodology of knit cotton fabric with Red 2 and Red 195 dyes.

The dyeing trials of original and treated samples were carried out with a liquor ratio of 1:20 in a rotary dyeing machine (Automatic Prototype, Model: A-12, AQUA, Guangzhou, China). The dye fixation temperatures for Red 2 and Red 195 were at 40 °C and 60 °C respectively. The dyeing processes are presented in Figure 2. During both dyeing processes, 40 g L^−1^ of NaCl was added as an electrolyte to promote dye exhaustion, and 10 g L^−1^ of Na_2_CO_3_ was added for dye fixation. Then, the dyed fabric was soaped in the rotary dyeing machine using a 2 g L^−1^ of detergent solution at a liquor ratio of 1:50 for 40 min at 95 °C.

#### 2.3.6. Dyeing Performance

The dye exhaustion percentage (E%), dye fixation rate (F%), and total dye fixation efficiency (T%) were calculated using Equations (1)–(3) respectively. The light absorbance value of dye solutions was calculated by a laboratory-scale Cary 100 UV-visible spectrophotometer (Agilent Technologies, Mulgrave, Melbourne Australia). The amount of absorbance was recorded at maximum wavelengths of 540 nm and 542 nm for Red 2 and Red 195, respectively.

(1) $$E\%=\frac{A_{0}-A_{1}}{A_{0}}\times 100\%$$

(2) $$F\%=(1-\frac{A_{2}}{A_{0}{-A}_{1}})\times 100\%$$

(3) T%=E%×F%×100%

where A_0_ and A_1_ indicates the light absorbances of the initial dye solution and the residual dye solution, respectively, and A_2_ is the light absorbance of the soaped solution.

#### 2.3.7. Color Uniformity

The K/S value (color strength) of the soaped dyed cotton fabrics was measured with a Datacolor 110 spectrometer (Datacolor International, Rotkreuz, Switzerland) at 20 random locations. The standard deviation value of the K/S (σ) was used to explore the color uniformity, and the lower the *σ* value, the better the color uniformity.

#### 2.3.8. Rubbing Colorfastness and Washing Colorfastness

Colorfastness to dry and wet rubbing and washing of the specimen were achieved as per ISO 105-X12:2011 with a crockmeter (Y571T, Ningbo Textile Instrument Factory, Ningbo, China) and ISO 105-C06:1997 (Test number: C2S) with a launderometer (SW-12, Ningbo Textile Instrument Factory, Ningbo, China), respectively. The colorfastness to washing was rated by evaluating the staining of the cotton fiber in the multifiber fabric by comparison with the ISO greyscale.

#### 2.3.9. Assessment of Fabric Softness

Fabric softness was assessed using a WOOL HandleMeter (Milspec Manufacturing, North Albury, Australia) [13]. The overall testing process was performed based on the draft test method. Before the test, each specimen was prepared into a circular shape and laid on the top surface of an orifice plate. Then, the knit sample was lowered automatically by a 453 g mass plate. Then, the sample was fully pushed through the orifice plate by a force rod. After that, the numerical values were recorded, and the hard/soft rate was determined, which ranged from 1 (extremely hard) to 10 (extremely soft). 

## 3. Results and Discussion

### 3.1. XRD Analysis of Cotton Fabrics

The XRD analysis was employed to investigate crystal allomorph and crystallinity change of the cellulose after each treatment. The FitYK 1.3.1 program was used to manage the diffraction background and to determine the area that occupied the peaks in the patterns. The background was treated in linear mode using stripe function from the program and the peaks were deconvoluted using the Gaussian peak fitting function. Afterwards, the crystallinity index (CI) was determined using Equation (4).

(4) $$\mathrm{CI}=\frac{I_{c}}{I_{c}+I_{a}}\times 100,$$

where I_c_ deotes the area of the crystalline domain and I_a_ for the amorphous domain area.

Table 1 summarizes the CI obtained from diffraction patterns of the original and treated cotton specimens in different protocols. The original sample consisted of a large volume of a highly ordered crystalline region with a CI of 78.12%. The CI decreased to 60.34%, and 58.43% after the CM and LA treatments. The CI further decreased to 41.23% and 38.87% after the sequential CM/LA and LA/CM treatments. It is noteworthy that the LA treatment offered a better effect on cellulose allomorph conversion than CM, either applied as an individual treatment or in combination with CM, which was mainly due to its low surface tension and smaller molecular size (3.11–3.99 Å) [14]. Thus, liquid ammonia was able to infiltrate crystalline quickly, while penetrating low-order areas and pores [14,15,16]. However, compared to alone CM and LA treatments, sequential treatment exhibited a better effect on the crystallinity modification and forming a high volume of an amorphous region within the cotton fiber because of their collaborative effect. The decreased crystallinity of the samples by CM, LA, CM/LA, and LA/CM was most likely due to the microfibril swelling, crystalline disruption, and the development of a new crystalline structure [16,17]. Following treatment with caustic solution and liquid ammonia, sodium ions and ammonia molecules penetrated through the microfibrils, leading to hydrogen bond breaking in both the crystalline and the amorphous regions. This contributed in fiber swelling and formation of Na-cellulose by CM treatment and NH3-cellulose formation by LA treatment, respectively [18,19]. The Na-cellulose I has subsequently been converted into Na-cellulose II and NH_3_-cellulose I into NH_3_-cellulose III. The new hydrogen bond networks in amorphous and crystalline areas with new patterns of cross-linking named cellulose II and cellulose III respectively were created when the liquid ammonia and NaOH solution had been removed from the cellulose complexes [19,20]. In addition, the cellulose microfibrils were difficult to recrystallize, which made for a smaller crystallinity in the treated cotton samples [14].

**Figure 3:**
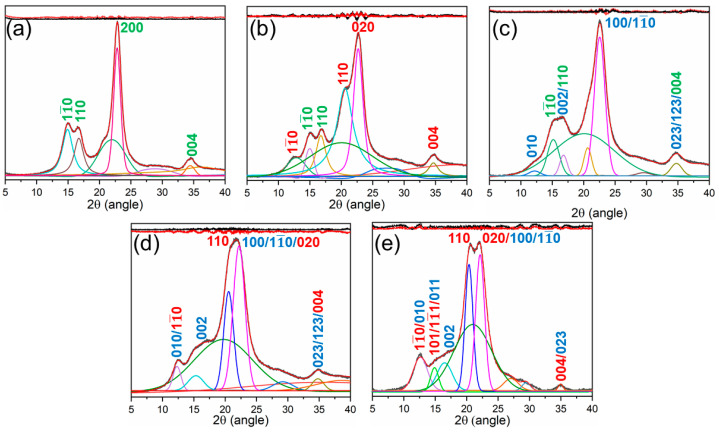
XRD analysis of (**a**) the untreated, (**b**) CM, (**c**) LA, (**d**) CM/LA, and (**e**) LA/CM samples with Miller indices: cellulose I (green labels), cellulose (red labels) and cellulose III (blue labels).

The diffraction patterns were further adopted to study the cellulose allomorph conversion by the different treatments. Figure 3 compares the diffraction pattern of samples obtained from the untreated and treated samples. Figure 3a shows a distinctive cellulose I pattern with intensive peaks of 2θ of 14.7, 16.5, 22.6, and 34.8°, which corresponds to the lattice planes (1$\bar{1}$0), (110), (200), and (400) were accordingly exhibited by the untreated samples [15,21,22]. In addition, the peaks contain a more complex geometry and a higher intensity, which causes large crystals to be present in the samples [23]. After either CM or LA treatment, it was found that the resultant diffraction pattern of sample corresponded to cellulose II or cellulose III allomorphs (Figure 3b,c). The pattern recorded after CM treatment revealed the peaks of (1$\bar{1}$0), (110), (020), and 004 lattice planes at 12.4, 20.5, 22.0, and 34.9°, respectively. These peaks suggest the transformation of cellulose I into cellulose II [16,18]. After LA treatment, the diffraction pattern exhibited the peaks of cellulose III allomorph at 2θ = 12.0, 14.9, 21.2, 29.8, and 35.4° assigned to the (010), (002), (100/1$\bar{1}$0), and (023/123) lattice planes, respectively [21,24]. Moreover, the peaks became broader and their intensities lowered, which suggests that the crystal size in the sample were decreased by these treatments. Interestingly, the diffraction patterns after either CM/LA or LA/CM treatment presented cellulose II and cellulose III allomorphic mixed lattices (Figure 3d,e) [15,25]. Both sequentially treated sample reveals the homogenously blended peaks of cellulose II lattice planes (1$\bar{1}$0, 110, 020, and 004) lattice planes and (010), (002), (100/1$\bar{1}$0), and (023/123) planes cellulose III allomorph, respectively. The peaks became wider and flatter after the sequential treatments (Figure 3d,e), which are most likely the result of a larger degree of defects in the cellulose crystal imparted by the combined effect of caustic solution and LA [15,25]. The changes in the diffraction spectrum have meant that cellulose allomorphs were converted from cellulose I to cellulose II and finally mixed configuration of cellulose III and II after CM/LA treatment. In contrast, after treatment with LA/CM, cellulose I changed into cellulose III and finally formed cellulose II and cellulose III mixed allomorphs. This ensured that the final samples consisted of both types of cellulose II and cellulose III allomorphs. The diffraction study suggested that the sequential treatments of CM/LA and LA/CM exerted a better effect on the new crystalline lattice formation, and decreased crystallinity than the samples treated with LA and CM independently.

### 3.2. FTIR Spectra

**Figure 4:**
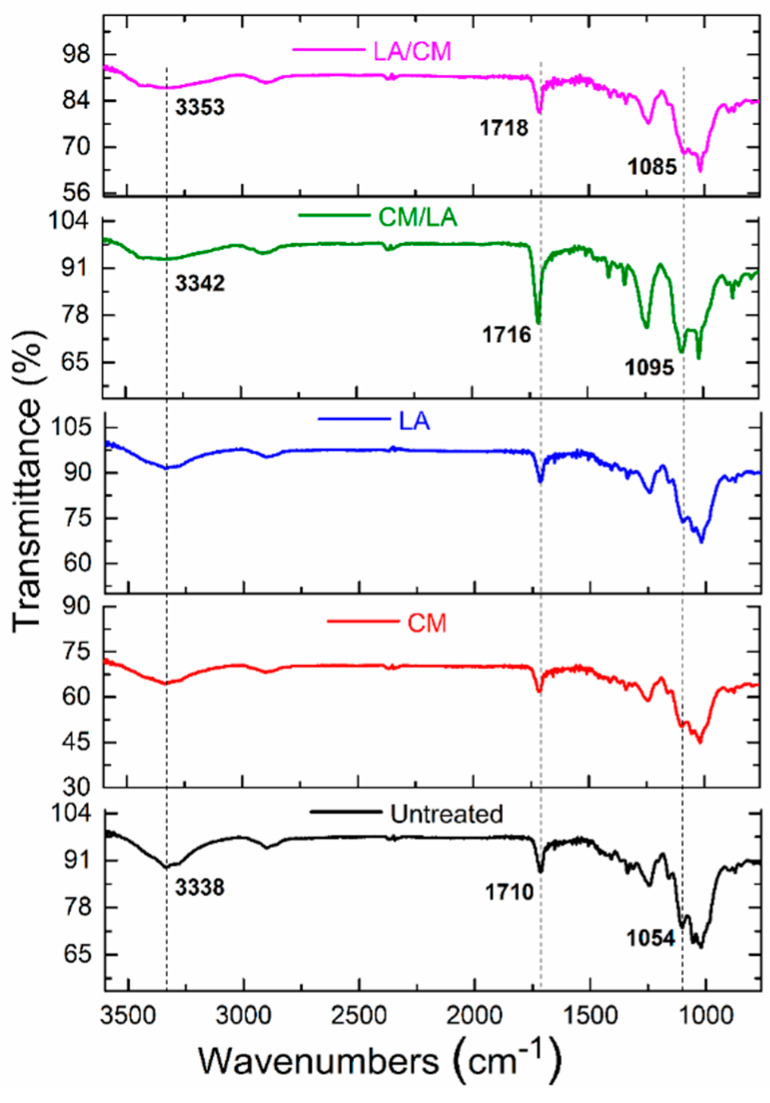
FTIR spectra of untreated and treated samples.

The chemical interactions between the untreated and sequentially treated samples were identified by FTIR spectra, as shown in Figure 4. The untreated, CM-treated, and LA-treated sample spectra exhibited strong absorbance peaks around 3000–3700 cm^−1^, which was attributed to the intramolecular hydrogen bonding between the hydroxyl groups [26]. However, the combined treatment samples (CM/LA and LA/CM) exhibited broader stretching vibration bands with higher wavenumbers, implying changes in their hydrogen bonds and the transformation of cellulose II and cellulose III from cellulose I after the sequential treatment [27]. A change was also noted in the absorption spectra from 1750 to 800 cm^−1^. The CM/LA-treated sample showed a strong absorbance band at 1095 cm^−1^, suggesting that its crystallinity was lower than the untreated sample. The FTIR was further applied to evaluate the effect of different sequential treatments on the hydrogen bonding intensity (HBI) of the cellulose macromolecules. The HBI is a ratio between 3336 and 1336 cm^−1^, indicating the degree of intermolecular regularity in the highly ordered crystalline region [28,29]. It was found that the untreated fibers had an HBI of ~1.53, while after CM and LA treatment, the HBI decreased to ~1.49 and ~1.43, respectively. The HBI of the samples further decreased to ~1.41 and ~1.34 after CM/LA and LA/CM, respectively. The decreased HBI values suggested that the highly ordered crystalline region was ruptured due to the swelling of the fibers and intramolecular irregularity increased because of the conversion process of the cellulose allomorph from cellulose I to cellulose II and cellulose III, respectively [30]. 

### 3.3. Thermal Performance

**Figure 5:**
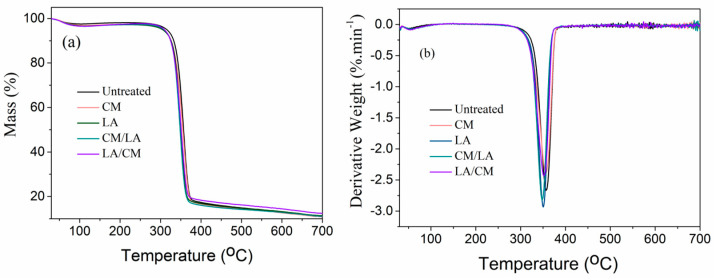
(**a**) TGA and (**b**) DTG curves of untreated and treated samples.

The thermal stability of the untreated and treated samples was determined by measuring the curves of TGA and DTG, as shown in Figure 5a,b. The untreated sample, i.e., pure cotton fabric, exhibited onset temperature at an initial stage (T_onset_, 356 °C), and the maximum weight loss percentage appeared at 374 °C in which the depolymerization behavior occurred by trans-glycosylation reactions [31]. Interestingly, it could be seen that the treated samples decompose rapidly at various temperature in comparison with the original sample. Moreover, the major weight loss begins slowly for both CM and LA treated cotton fabrics than the untreated sample. These were ascribed to the presence of a greater amount of amorphous cellulose, which underwent faster thermal decomposition than the crystalline phase [32]. All of these data indicate that the LA-treated sample showed less thermal stability, as confirmed by the TGA and DTG curves, which corresponds to the previous report [33]. Although the T_max_ of untreated cotton fabric was higher among all samples, the amount of char residue was lower than that of treated fabrics (Table 2). The percentage char for the LA/CM-treated sample showed highest, meaning that this sample will have satisfactory flame retardancy properties [34]. Mainly, it was found that there are no substantial changes observed in their TGA patterns (untreated and treatment) by applying sequential pre-treatment technologies such as liquid ammonia and caustic mercerization. However, it must be mentioned that pre-treated samples exhibited a higher thermal stability compared to the untreated sample; this indicates that these treatments were efficient in the removal of compounds [35] such as hemicellulose and lignin. Overall, the necessity of the pre-treatments of mercerization of the cotton fabric before the dyeing process can be underlined to obtain better physicochemical properties [36].

### 3.4. Wicking Properties

**Figure 6:**
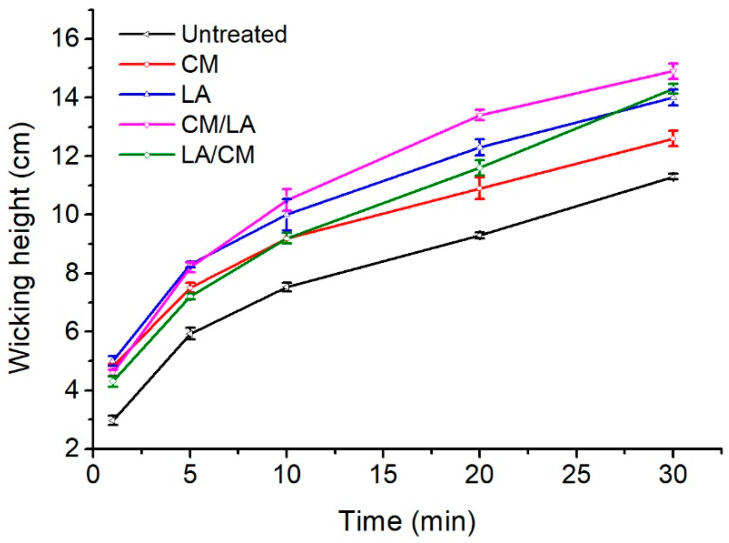
Wicking heights of the untreated and treated cotton fabrics at different test times.

Wicking occurs when a liquid flows through capillary channels in fabrics due to capillary forces [37]. The wicking heights of cotton fabrics treated by CM, LA, CM/LA, and LA/CM are displayed in Figure 6. After 1 min, the wicking height of the treated samples became higher than the control sample, and the difference in the wicking heights of the treated samples was small. Upon increasing the test time, all wicking heights gradually increased, and the variances between the treated samples expanded. All treatments improved the wicking properties of the fabric within 30 min because the wicking heights increased from 11.2 cm in the original sample to 12.6, 14.0, 14.9, and 14.3 cm in the CM-, LA-, CM/LA-, and LA/CM-treated fabrics, respectively. The increase was due to changes in the hydrophilicity of the cotton fiber [38]. The number of hydroxyl groups in cotton fibers rose as CM, LA, and their combinations lowered the crystalline zone and improved the amorphous region. This resulted in an improved cotton fiber hydrophilicity. These treatments also enlarged the cumulative accessible pore volume [11], which manifested as increased water adsorption (wicking). Furthermore, the CM treatment expanded the fiber pores, but the LA treatment compacted the fiber pores, which decreased the wicking height of the CM-treated fabric compared with others [39]. 

### 3.5. Treatment Influence on Dyeing Properties

**Figure 7:**
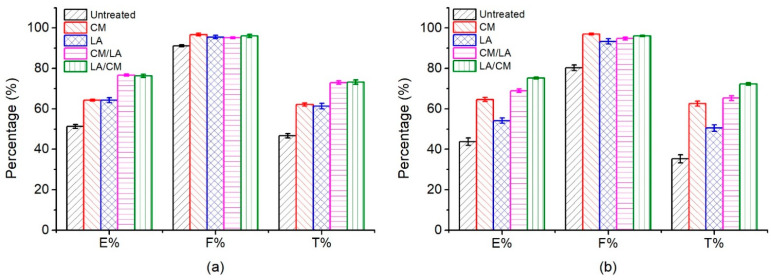
E%, F%, and T% of the dyeings of untreated and treated cotton fabrics using (**a**) Red 2 and (**b**) Red 195.

The untreated and treated fabrics were dyed with Red 2 and Red 195, and the E%, F%, and T% values shown in Figure 7 indicate that all treatments improved the dye exhaustion percentage. During the dyeing of cotton fabrics, dyes only reach the amorphous region, which typically means that a larger amorphous region (i.e., lower crystallinity) increases dye exhaustion [40]. Since all treatments decreased the crystallinity, the E% values increased after each treatment [41]. An increase in the amorphous region was accompanied by a higher cumulative accessible pore volume [11], which increased E% because the dyes migrated from the fiber interior to the surface via pores.

The dyeings of CM-treated and LA-treated cotton fabrics with Red 2 (Figure 7a) showed nearly the same E% values, which were 64.25% and 64.33%, respectively. For the CM/LA treated and LA/CM treated samples, the E% increased to 76.68% and 76.27%, respectively, from 51.30% of the untreated fabric. This indicates that the combined treatments further improved the dye exhaustion ability of the cotton fabric dyed with Red 2, compared with the single treatment of CM or LA. In contrast, for the treated cotton fabric dyed with Red 195 (Figure 7b), the change in E% was different from the fabric dyed with Red 2. The E% value of the fabric treated by CM increased to 64.64% from 43.79% (control fabric), but it was only 54.16% for the fabric treated by LA. This shows that the CM treatment had better dye exhaustion than the LA treatment when the cotton fabric was dyed with Red 195, which is consistent with the previous research [42]. Moreover, the LA/CM-treated specimen showed a higher E% (75.25%) than the CM/LA-treated specimen by (68.97%). The dye exhaustion results of Red 195 are similar to the vales in the paper [10]. This result implies that the treatment sequence affects the dye exhaustion of cotton fabric dyed with Red 195. 

The pores inside cotton fabrics are categorized as either small, medium, or large [24], which means that small molecules can enter all pores, but large molecules cannot enter small or medium pores. The CM, LA, CM/LA, and LA/CM treatments changed the pore sizes and increased the cumulative accessible pore volume. However, CM treatment expanded the pore size, while LA treatment contracted the pore size [43]. During reactive dyeing, the dyes that adsorbed onto the fiber surface migrated inside the fibers through the pores; thus, the pore sizes and the dye molecular size affected the dye migration and exhaustion. The molecular weight of Red 2 is 615.32 g mol^−1^ and Red 195 is 1136.28 g mol^−1^. Since Red 195 is larger than Red 2, during the knit cotton fabric dyeing, the E% of Red 2 was higher than that of Red 195. After the CM treatment, the E% of Red 2 was similar to the E% of Red 195, but after the LA treatment, the E% of Red 2 was approximately 10% higher than that of Red 195 because of the smaller pore size [11].

Interestingly, the CM/LA-treated and LA/CM-treated cotton fabrics dyed with Red 2 had the same E% values, because the Red 2 molecule was small enough to migrate through the small size pore of cotton fiber, which is consistent with the wicking results. However, different E% values were observed between the CM/LA and LA/CM treatments for the fabric samples dyed with Red 195. The pore sizes expanded after the first CM treatment, but some pores contracted to small pores after the second LA treatment, which inhibited the Red 195 dye exhaustion into the CM/LA-treated cotton fabric. For the LA/CM-treated fabric, after the first LA treatment, the cumulative accessible pore volume increased, and the pores contracted; however, the pores expanded after the subsequent CM treatment, showing a high E% [11]. 

In general, the combined treatments of LA/CM or CM/LA had better dye exhaustion than the single treatment of CM and LA in dyeing of cotton fabrics [6]. The sequential treatments with a final CM process promoted the dye exhaustion rate of the cotton fabrics dyed with both small and large dyes, but the combined treatments with a final LA process more effectively promoted the exhaustion of the smaller molecular size dye (Red 2) than the larger dye (Red 195). The results are coincident with the dyeing findings [11].

Dye fixation is a chemical reaction to form covalent bonds between reactive dyes and cellulosic fiber. Under their respective fixation conditions, the treated fabrics showed a higher F% value compared to the untreated fabric. The treatments decreased the cotton fiber crystallinity and produced more amorphous regions, which means that more hydroxyl groups were produced [44,45] that could covalently bond with the dyes. The total dye fixation efficiency (T%) relates the dye exhaustion to fixation and is determined by the percentage of the fixed dye mass in the fibers to the initial dye mass. Therefore, it is important to select small molecular size reactive dyes in the dyeing of knit cotton fabric finally pre-treated by LA process, which can extremely utilize its improved dyeability, i.e., saving dye consumption. 

### 3.6. Color Uniformity

In Table 3, the standard deviations of the K/S values (σ) indicate that all dyed samples have uniform shades. The K/S values and the color uniformity of the LA/CM-treated cotton fabrics dyed with Red 2 and Red 195 were better than the CM/LA-treated cotton fabrics. This indicates that the color shade and the color uniformity of the dyed cotton fabric were influenced by the LA treatment. 

### 3.7. Colorfastness to Rubbing and Washing

The colorfastness to rubbing and washing of the treated dyed fabrics are listed in Table 4. In general, all colorfastness grades were good, which may have been owing to the building of covalent interactions between the reactive dyes and the cellulosic fibers, in combination with a thorough washing procedure. 

### 3.8. Treatments Influence on Fabric Softness

**Figure 8:**
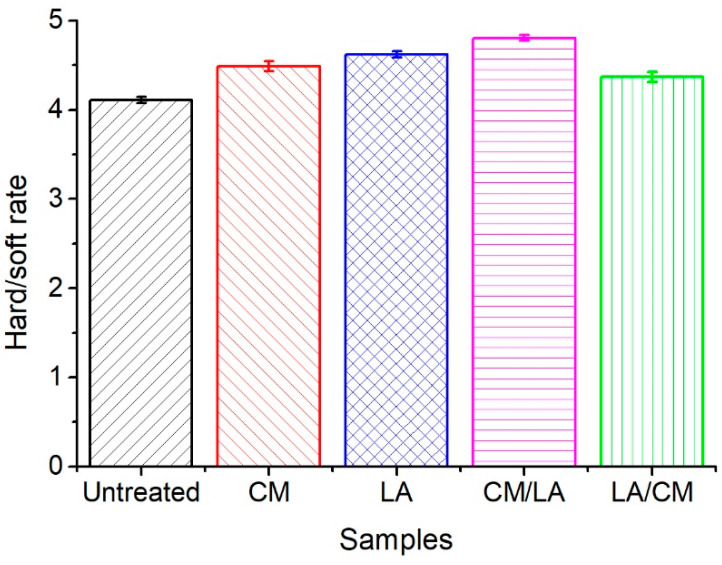
Effect of CM, LA, CM/LA, and LA/CM pretreatments on soft handle of knit cotton fabric.

The softness handle of the control and pre-treated cotton fabrics dyed with Red 2 was measured using a WOOL HandleMeter, and the results are shown in Figure 8. The previous report [9] states that CM treatment on woven cotton fabric brings about a stiff handle, but it is different on CM treatment of knit cotton fabric. A higher value in Figure 8 indicates that the sample has a softer handle. The softness of the dyed untreated cotton fabric (dyed control) was 4.1074, and all treated fabrics showed considerably better handle properties than the dyed control fabric. The fabric treated by CM treatment exhibited a handle rating of 4.4867, but the enhancement was lower than that of the LA treated fabric, which exhibited a handle rating of 4.6218, i.e., the treated fabric was soft. After the combined CM and LA treatments, the treatment order significantly affected the softness handle. The cotton fabric treated by CM/LA had the highest softness handle, and the fabric treated by LA/CM showed the lowest softness of these treated cotton fabrics, which were 4.8051 and 4.3675, respectively. Therefore, it seems that the fabric finally treated by LA showed a better softness handle, and this conclusion is supported by the published papers [6,7].

According to the XRD analyses of the cotton fibers, the lower CI values possibly contributed to the improved handle values since all treatments changed the internal structure of the cotton fibers [10]. The soft handle also tended to increase as the CI value of the treated cotton fiber decreased, except for the LA/CM treatment; thus, it can be summarized that the CM/LA-treated cotton fabric exhibited the best soft handle property. 

## 4. Conclusions

Various physicochemical properties of knit cotton fabrics were enhanced by sequential liquid ammonia and caustic mercerization pretreatments. The two-stage process changed the crystallinity of the cellulose with mixed configurations of cellulose II and cellulose III, and the CI values of the sequentially treated samples decreased. The CM/LA-treated fabric exhibited considerably more wicking among all samples, and the results also demonstrated that a larger pore size after CM treatment decreased the wicking. The results showed that the sequentially treated samples (CM/LA and LA/CM) showed better thermal stability than the individual treatment. Reactive Red 2 offered better dyeability than Reactive Red 195, although the latter was applied at a much higher temperature, which suggests that dyes with higher molecular weights are ineffective after liquid ammonia treatment. The colorfastness was good after the two-stage treatment. Moreover, the soft handle property of treated fabrics was enhanced compared with the untreated fabric, and the CM/LA treated fabric exhibited the best soft handle. This research has shown that a two-stage CM/LA treatment provides a potential treatment method for improving various physicochemical properties of cotton fabrics. The CM/LA-treated cotton fiber can be used for deep shade dyeing requirements, accompanied by a mercerization performance, especially with a soft handle.

## Figures and Tables

**Table 1:** The crystallinity index data.

Sample	Original	CM	LA	CM/LA	LA/CM
CI (%)	78.12	60.34	58.43	41.23	38.87

**Table 2:** Thermal stabilities of samples.

Specimen	Temperature at Initial Cleavage (T_onset_, °C)	Temperature at Maximum Degradation (T_max_, °C)	Char Residue at 700 °C (wt.%)
Untreated	356	374	10.91
CM	354	370	10.96
LA	352	365	11.38
CM/LA	347	369	11.05
LA/CM	350	367	12.38

**Table 3:** K/S and color uniformity values.

Dyed Fabric	Index	Untreated	CM	LA	CM/LA	LA/CM
Red 2	K/S	4.58	5.87	5.99	6.29	6.73
	σ	0.0469	0.0539	0.0500	0.0400	0.0922
Red 195	K/S	4.03	5.91	4.90	6.09	6.49
	σ	0.0300	0.2179	0.1887	0.2472	0.2425

**Table 4:** Rubbing and washing colorfastness (staining) values.

Treatment	Rubbing Fastness (Dry/Wet)		Wash Fastness	
	Red 2	Red 195	Red 2	Red 195
Original	5/4-5	5/4-5	5	4-5
CM	5/4	5/4	5	4-5
LA	5/4-5	5/4-5	5	4-5
CM/LA	5/4-5	5/4-5	5	4-5
LA/CM	5/4-5	5/4-5	5	5

## Data Availability

The datasets generated during the current study are available from the corresponding author on reasonable request (Yingjie Cai, Y.C.).
